# Injectable and Spatially Patterned Microporous Annealed Particle (MAP) Hydrogels for Tissue Repair Applications

**DOI:** 10.1002/advs.201801046

**Published:** 2018-10-11

**Authors:** Nicole J. Darling, Elias Sideris, Naomi Hamada, S. Thomas Carmichael, Tatiana Segura

**Affiliations:** ^1^ Department of Chemical and Biomolecular Engineering University of California, Los Angeles 420 Westwood Plaza Los Angeles CA 90095 USA; ^2^ Department of Neurology David Geffen School of Medicine University of California, Los Angeles 621 Charles Young Drive Los Angeles CA 90095 USA

**Keywords:** injectable, patterning, porous scaffolds, skin, stroke

## Abstract

Spatially patterned hydrogels are becoming increasingly popular in the field of regenerative medicine and tissue repair because of their ability to guide cell infiltration and migration. However, postfabrication technologies are usually required to spatially pattern a hydrogel, making these hydrogels difficult to translate into the clinic. Here, an injectable spatially patterned hydrogel is reported using hyaluronic acid (HA)‐based particle hydrogels. These particle hydrogels are sequentially loaded into a syringe to form a pattern and, once injected, they maintain the pattern. The applicability of this hydrogel in a wound healing skin model, a subcutaneous implant model, as well as a stroke brain model is examined and distinct patterning in all models tested is shown. This injectable and spatially patterned hydrogel can be used to create physical or biochemical gradients. Further, this design can better match the scaffold properties within the physical location of the tissue (e.g., wound border vs wound center). This allows for better design features within the material that promote repair and regeneration.

The extracellular matrix (ECM) is a heterogeneous network of biopolymers including complex proteins and sulfated and nonsulfated glycosaminoglycans that provide mechanical support and organization to the tissue, and spatiotemporal presentation of chemical cues.^1^ Collectively, these features control cell growth, migration, and differentiation, which lead to tissue homeostasis. After injury, the ECM is broken down by mechanical forces and proteases released by inflammatory cells.^2^ While in normal skin wound healing, temporary matrix degradation ensures that the wound is clean and ready for new tissue deposition, in nonhealing chronic wounds, matrix degradation limits the physical and chemical support provided by the surrounding ECM to promote new tissue deposition.^3^, ^4^ In the brain, injury causes not only matrix degradation, but also the formation of an impermeable astrocytic scar that engulfs the injured area.^5^ While this prevents further brain damage, it also prevents new matrix deposition in this area. In an effort to accelerate wound closure and promote healing in nonhealing skin or brain wounds, the field of tissue repair has investigated the use of artificial ECM matrices, which recapitulate aspects of the natural ECM, and can be utilized by residing cells as a scaffold for tissue growth.^6^ The goal is to recapitulate sufficient aspects of the matrix to promote tissue repair of such promoting infiltration of residing cells that can build their own native ECM as the temporary synthetic ECM degrades away.

Over the past several decades, polymeric hydrogels containing physical and bioactive cues have been developed as ECM mimics.^7^ Specifically, injectable hydrogels which form via a sol–gel transition are highly translatable as they can be injected and take on the form of any wound or cavity before complete gelation in situ. However, most injectable hydrogels are physically and chemically homogeneous, a property not observed in the native ECM.^6^ Physical or chemical gradients are often necessary to recruit cells to a specific location like a wound or stroke cavity.^8^, ^9^, ^10^, ^11^ An Ac‐RGDSPGERCG‐NH2 (RGD) gradient has previously been shown to direct cell alignment and migration in vitro.^12^ Yet the most common method of incorporating either a physical or chemical gradient in a hydrogel is by using photopolymerization, which results in a hydrogel that cannot be injected and is significantly limited in its use in the clinic.^13^, ^14^, ^15^, ^16^, ^17^ The development of an injectable hydrogel with defined gradients still poses a major challenge in the field.

We previously engineered the first class of injectable synthetic porous hydrogels, termed microporous annealed particle (MAP) hydrogels.^18^, ^19^, ^20^ MAP hydrogels are granular materials constructed from micrometer‐sized hydrogel particles that are annealed to each other postinjection. These granular hydrogels contain an interconnected micrometer‐sized porous network due to the uneven packing of the building blocks. Although the use of granular MAP scaffolds in tissue repair and regeneration is just beginning, their ability to accelerate wound healing^18^ and reduce inflammation^19^ makes them ideal artificial ECM scaffolds for tissue repair applications. However, so far, the building blocks have been uniformly mixed, generating a random configuration of building blocks. Herein, we show that spatial patterning of granular hydrogel materials can be achieved by taking advantage of jammed layered particle structures, which when injected at appropriate flow rates, retain their layered structures after injection and anneal into the subcutaneous space, skin wounds, and brain stroke wounds, demonstrating the versatility of the approach.

Microgel (µgel) hydrogel building blocks with ≈100 µm diameter were generated using a flow focusing microfluidic device as previously described.^20^ µgels were labeled during fabrication with three different fluorophores (555, 488, 647 nm shown as red, green, white, respectively) such that the layers could be visualized with fluorescent confocal microscopy. The µgels also contained two peptides which are substrates of factor XIIIa (FXIIIa), such that annealing can occur through this enzymatic reaction as previously demonstrated.^18^, ^19^, ^20^ We first tested if injecting sequential granular MAP scaffolds next to each other would allow distinct layers to be formed without mixing. In vitro, µgels in equal volumes were injected into a poly(dimethyl)siloxane (PDMS) mold (**Figure** **1**A) from left to right in rapid succession (green, followed by red, followed by far‐red µgels). ImageJ analysis confirmed an intensity profile from left to right, showing a peak in green pixels first, followed by red pixels, and white pixels last, indicating that the µgels retain their position after injection. Analysis of the percent of each color in each layer reveals that a large majority of µgels for each of the layers contained the color injected in that layer, with 89% green µgels found in the first layer injected, 80% of red µgels found in the second layer injected, and 69% of far‐red µgels found in the last layer injected. The red region is in equal contact with both the green and white regions and was found to contain equal percentage of white and green µgels. For both the green and far‐red regions, very few of the white and green µgels were found in each other's regions because the white and green regions are not in contact with each other. These data show that in vitro a straight left to right pattern could be achieved through sequential injections followed by annealing. This linear pattern can be useful in vitro to study cell migration through different layers. Further, this strategy of creating layers is not limited to just three equal volume layers as more layers can be created.

**Figure 1:**
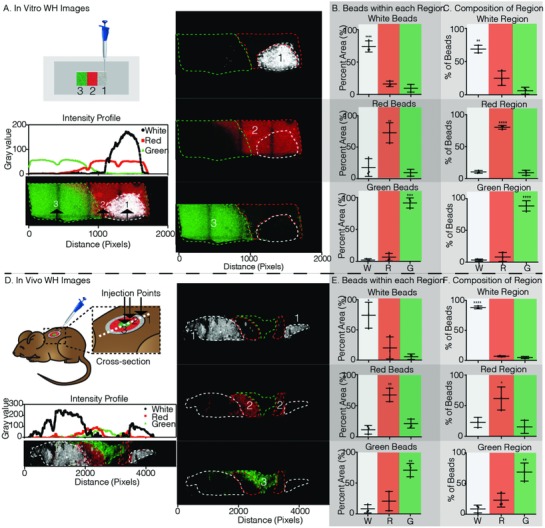
Wound healing in vitro and in vivo in situ patterning. A) Schematic of patterning method pipetting one color µgel at a time into a rectangular glass bottom PDMS well. Fluorescent images taken on the Nikon C2 analyzed in ImageJ to generate the intensity profile plot of each color µgel. The dashed lines indicate each color region. The numbers indicated the order of injection from the pipette. B) Percent of the total µgels of a given color within its respective color region is 74.15%, 72.74%, and 91.33% for white, red, and green, respectively. C) Color region µgel composition is dominated by the region color as 68.84%, 80.34%, and 88.59% of white, red, and green µgels within the white, red, and green regions. D) Schematic of patterning method pipetting one color µgel at a time, outside to inside, into a 6 mm full thickness dermal wound on the back of a mouse. On day 3, the tissue was collected, fixed, cut in half (depicted by the dashed white line), and imaged on the Nikon C2. The intensity profile plot of each color µgel along the cross‐section was generated. The dashed lines indicate each color region. The numbers indicated the order of injection from the pipette. E) Percent of the total µgels of a given color within its respective color region is 74.19%, 67.33%, and 70.97% for white, red, and green, respectively. F) Color region µgel composition is dominated by the region color as 88.23%, 61.72%, and 68.69% of white, red, and green µgels within the white, red, and green regions. Tukey's multiple comparison test (*p* < 0.05). Unless specified otherwise, *N* = 3.

Next, we tested our patterned hydrogel in an in vivo skin wound model. A biopsy punch was used to create a cylindrical skin wound on the backs of the mice (≈6 mm diameter × 1 mm high). Similar to the sequential injection strategy used to create side‐by‐side layers, here multiple injections were used to create a radial pattern. The first layer injected was the outmost layer and the last was the center (Figure 1D). Three days post layered gel injection, the skin was cut in half and imaged along the center using confocal microscopy. As expected, a radial pattern could be observed with white µgels on both the ends, red µgels adjacent to them, and green µgels in the center. An intensity profile from left to right quantitatively defined two white pixel peaks on the edges, one green pixel peak in the middle, and two red pixel peaks between the white and green peaks. Similar to the in vitro data, three clear regions are observed with 88% of white, 62% of red, and 69% of green µgels located in the white, red, and green regions, respectively (Figure 1E,F). The middle region (red µgels) is the most heterogeneous given it is the middle layer in contact with both the other regions, the layer is still comprised of a majority of red µgels (61.72 ± 18.84%). Together, these data show that a radial pattern consisting of distinct layers can successfully be achieved in vivo through sequential injections of MAP µgels.

For many applications such as injection into the subcutaneous space or in the brain, the desired location of the layered scaffold is not as easily accessible as it was in the wound healing model. For these situations, it is necessary to obtain layers with a single injection. We tested our hypothesis that layers of MAP µgels could be formed through a single injection if the syringe is sequentially loaded with the desired layers. 15 µL of each colored particle was sequentially pipetted up to obtain 45 µL of total gel in the pipette and then back‐loaded into a syringe to maintain the same pattern (**Figure** **2**A). A controlled syringe dispenser was then used to inject the hydrogel between two coverslips from the side, imitating a subcutaneous (Sub‐Q) injection to obtain a radial pattern. The resulting hydrogel scaffold was imaged by confocal microscopy and observed to indeed have a radial spatial pattern. Since the white µgels were pulled up first, they came out of the syringe last, while the red µgel were pulled up last and were the first to come out of the syringe. The pattern in Figure 2A shows that the red µgels were radially “pushed” outward by the green µgels which in turn were then radially “pushed” outward by the white µgels, creating three distinct layers within a single hydrogel scaffold with little mixing between the layers. Although the same volume of each color gel was injected, the outer surface occupied by each layer is variable, resulting in layers with different thicknesses. If equal layer thickness was desired, the volume of each injected gel would have to scale with the outer surface area occupied by that layer color.

**Figure 2:**
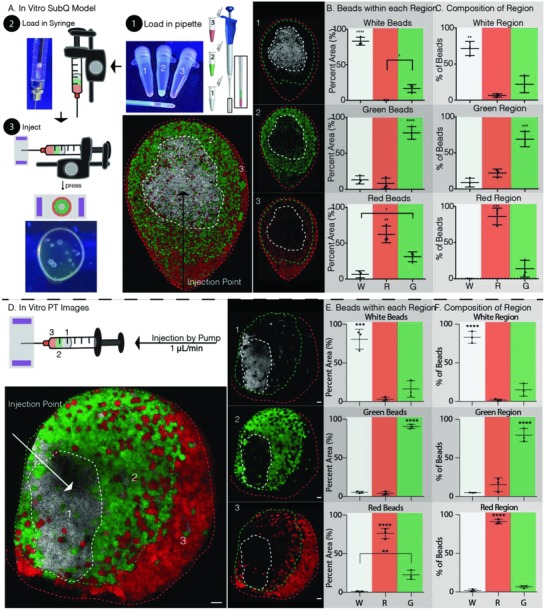
In vitro patterning method for Sub‐Q injection and PT stroke model. A) Schematic of patterning method by 1) loading the pipette in the order indicated by increasing number, 2) back‐loading the syringe, which reversed the order of injection, and 3) injecting between two glass slides by repeatably pressing the controlled syringe dispenser. The dashed lines indicate each color region. The numbers indicated the order loaded into the pipette. B) Percent of the total µgels of a given color within its respective color region is 83.21%, 62.26%, and 78.53% for white, red, and green, respectively. C) Color region µgel composition is dominated by the region color as 71.59%, 86.04%, and 69.03% of white, red, and green µgels within the white, red, and green regions. D) Schematic of patterning method using the same steps one and two as in part (A) but injecting between two glass slides using a pump at 1 µL min^−1^. The dashed lines indicate each color region. The numbers indicated the order loaded into the pipette. E) Percent of the total µgels of a given color within its respective color region is 80.79%, 76.29%, and 90.67% for white, red, and green, respectively. F) Color region µgel composition is dominated by the region color as 83.22%, 91.17%, and 79.55% of white, red, and green µgels within the white, red, and green regions. Tukey's multiple comparison test (*p* < 0.05). Unless specified otherwise, *N* = 3. Scale bars = 100 µm.

Upon analysis of the various layer distributions using ImageJ software analysis (Figure 2B,C), it was found that the majority of each colored µgel type was found within its own layer, without significant spreading to adjacent layers. Specifically, 72% of white, 69% of green, and 86% of red µgels were found in the white, green, and red layers, respectively. Moreover, it was observed that the majority of mixing occurred from the direct neighboring layering. As a result, very few white µgels were pushed to the outer red layer and similarly, very few red µgels remained in the white region.

Next, to model injection into a stroke cavity, the same two coverslip model was used, however, a syringe pump with a continuous injection rate of 1 µL min^−1^ was used to inject the hydrogel (a rate previously used in vivo).^19^ Here, the green µgels exited the syringe first, forming the outer layer, followed by the red µgels forming the middle layer, with the white µgels exiting last, forming the inner layer (Figure 2D). As before, ImageJ software was used to analyze the resulting images obtained from confocal imaging. The obtained results were improved to those obtained for in vitro subcutaneous injections (Figure 2A), showing that using a slower injection rate compared to a bolus injection results in less mixing and stronger patterns (Figure 2E,F). It was observed that 83% of white, 80% of red, and 91% of green µgels are in the white, red, and green regions, respectively. This shows that strong layering can still be achieved in the middle layer which is in contact with both the inner and outer layers. In fact, decreasing the injection rate improved the layering in the middle layer from 69.03% to 79.55% of all red µgels remaining within the middle red layer.

After the in vitro data modeling, a subcutaneous injection yielded positive results, we tested our patterned hydrogel in vivo. We followed the same procedure to load the syringe as in vitro and we injected subcutaneously into a mouse (**Figure** **3**A). The skin was collected after three days, fixed using paraformaldehyde, and imaged. Here, we did not notice a radial pattern like we did in vitro, likely as a result of the angled injection into a defined space. However, distinct white, red, and green regions could still be observed. Upon analysis of the layers, it was similarly shown that 65% of white, 69% of green, and 55% of red µgels were found in the white, green, and red layers, respectively. (Figure 3B,C). It was also found that in the inner white region, the majority of the mixed µgels were from the neighboring green region, while in the middle green region, a similar number of white and red µgels were found mixed in. Finally, in the outer red region, the majority of µgels mixed in were from the middle green region, similar to the results found in vitro. With these results, we are able to achieve a spatially patterned injectable hydrogel with three distinct regions in vivo using a subcutaneous implant model.

**Figure 3:**
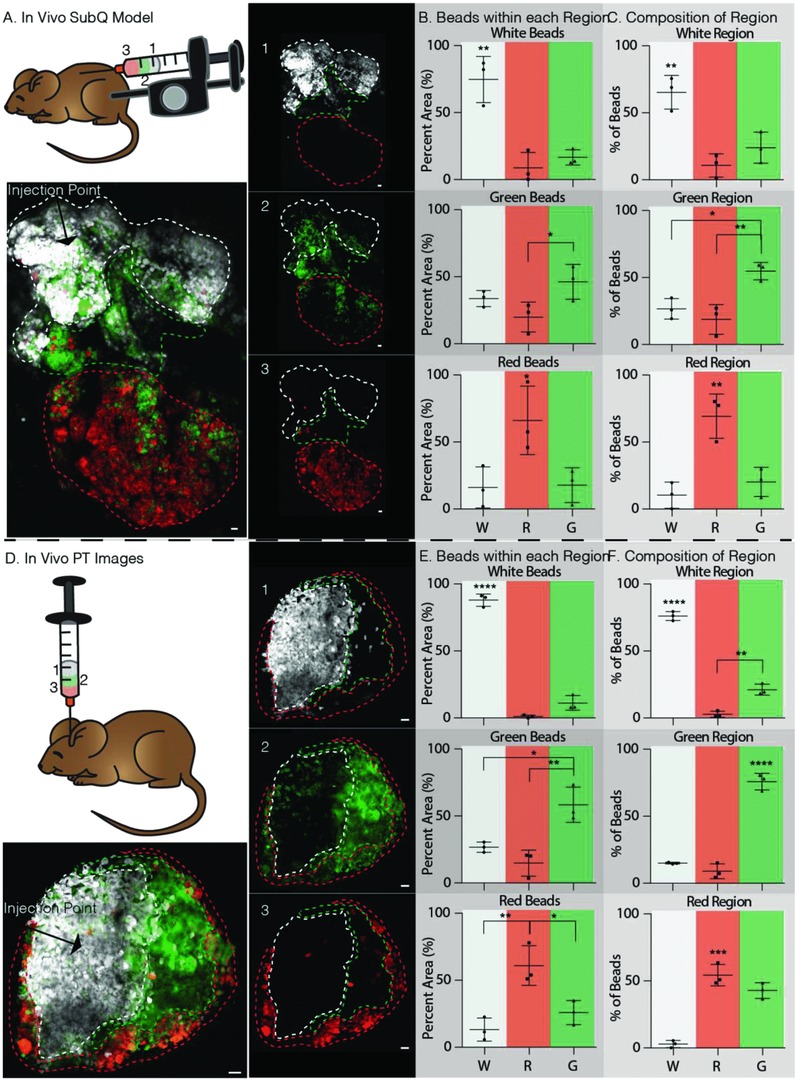
In vivo patterning method for Sub‐Q injection and PT stroke model. A) Schematic of patterning method using the same steps one and two as in Figure 2A and then injecting into the subcutaneous layer of the mouse hind skin by repeatably pressing the controlled syringe dispenser. On day 3, the tissue was collected and imaged on a glass slide, dermal layer up. The dashed lines indicate each color region. The numbers indicated the order loaded into the pipette. B) Percent of the total µgels of a given color within its respective color region is 74.69%, 66.11%, and 46.25% for white, red, and green, respectively. C) Color region µgel composition is dominated by the region color as 65.29%, 69.30%, and 54.76% of white, red, and green µgels within the white, red, and green regions. D) Schematic of patterning method using the same steps one and two as in Figure 2A and then injecting into a PT‐stroked mouse at 1 µL min^−1^. The dashed lines indicate each color region. The numbers indicated the order loaded into the pipette. E) Percent of the total µgels of a given color within its respective color region is 87.86%, 60.98%, and 58.42% for white, red, and green, respectively. F) Color region µgel composition is dominated by the region color as 76.11%, 54.29%, and 75.90% of white, red, and green µgels within the white, red, and green regions. Tukey's multiple comparison test (*p* < 0.05). Unless specified otherwise, *N* = 3. Scale bars = 100 µm.

Finally, we evaluated the in situ patterning in a mouse stroke model using a single, controlled injection of multiple MAP subunits. A photothrombotic (PT) stroke model to cause ischemia in the motor cortex was used to form a stroke cavity. Five days post stroke induction, the patterned hydrogel (6 µL total) was injected at 1 µL min^−1^ and allowed to anneal. Two weeks later (a time point used previously for tissue analysis^19^), the brains were collected for tissue processing and cryosectioning. Each brain sample was sectioned until the middle of the stroke lesion containing the largest hydrogel cross‐sectional area could be observed. The remaining brain was imaged under a confocal microscope (Figure 3D). Interestingly, unlike what was observed for the in vivo subcutaneous injections, a radial pattern was observed within the stroke cavity. Once again, the layers are of different thicknesses because the same volume of each color subunit was injected and in a radial pattern, the outer surface area occupied by each layer is different. Distinct layering was observed with the inner and middle regions, white and green, respectively. The outer red region was more mixed containing both red and green µgels. Specifically, 76% of white, 76% of green, while only 54% of red were observed in the white, green, and red regions, respectively (Figure 3E,F). We likely do not see as much of a separation of the green and red µgels in the green region because of the thinness of the green region. Regardless, we do observe excellent layering in the white and green regions, and a majority separation in the red region.

Similar to wound healing, we have previously shown that MAP gel can reduce inflammation and recruit neural progenitor cells to the stroke. However, the inclusion of a physical or biochemical pattern to better guide cells to the middle of the stroke lesion is predicted to further improve the biological response and lead to better stroke repair.^19^ Here, we showed that it is possible to create a pattern using different colored MAP subunits with a single injection or by sequential injection into an open wound. Given the tenability of these subunits, additional studies can evaluate the impact of brain regeneration or skin wound healing following stroke using various MAP subunits containing different stiffnesses or densities of peptides (i.e., RGD, etc.) or growth factors (i.e., vascular endothelial growth factor (VEGF), brain‐derived neurotrophic factor (BDNF), etc.) to further guide neuronal or keratinocyte cell migration. Stiffness gradients can be created by changing the stiffness of each µgel layer which have shown to promote cellular migration with various cell types.^21^, ^22^ Growth factor gradients can similarly be created by encapsulating different concentration of growth factor in each layer and also have been shown to significantly affect cellular behavior.^23^, ^24^

Here, we engineer MAP hydrogels into injectable, gradient hydrogels in the skin and brain. To the best of our knowledge, this is the first example of an injectable, porous, gradient hydrogel with the potential for use in vivo. It was shown that a variety of building blocks can be used to create MAP hydrogels and the spatial presentation of the building blocks can be controlled to create layers. Both sequential injections and single injections can be used to establish gradients of different colored MAP subunits in vitro and in three models in vivo, wound healing, subcutaneous space, stroke cavity. Only a low degree of spreading was observed between adjacent layers, that was further improved through controlled injection rates via syringe pumps. We believe that this platform can have widespread use in the field of regenerative medicine as it can be adapted to multiple organs. Moreover, a variety of gradients can be created using this hydrogel technique as the MAP subunits are highly tunable.

## Experimental Section

*Hyaluronic Acid Modification*: Hyaluronic acid (HA) was functionalized with an acrylate group using a previously described two‐step reaction.^25^ Briefly, HA (60 000 Da, Genzyme Corporation, Cambridge, MA) (2.0 g, 5.28 mmol) was dissolved in water mixed with adipic dihydrazide (ADH, 18.0 g, 105.5 mmol) with 1‐ethyl‐3‐(dimethylaminopropyl) carbodiimide hydrochloride (EDC, 4.0 g, 20 mmol) with pH adjustment to 4.75. This mixture reacted overnight to form hydrazide‐modified hyaluronic acid (HA‐ADH). Purification via dialysis (8000 molecular weight cut‐off (MWCO)) was completed in deionized water for two days. The HA‐ADH was then flash‐frozen and lyophilized. HA‐ADH (1.9 g) was dissolved in 4‐(2‐hydroxyethyl)‐1‐piperazine ethanesulfonic acid (HEPES) buffer (10 × 10^−3^ m HEPES, 150 × 10^−3^ m NaCl, 10 × 10^−3^ m ethylenediaminetetraacetic acid (EDTA), pH 7.4) and mixed with *N*‐acryloxysuccinimide (NHS‐AM, 1.33 g, 4.4 mmol) and reacted overnight. Purification via dialysis against deionized water for two days was completed, and HA–acrylate was flash‐frozen and lyophilized. The product was analyzed with 1 H NMR (D20) and the percent modification (14%) was determined by dividing the multiplet peak at δ = 6.2 (*cis* and *trans* acrylate hydrogens) by the singlet peak at δ = 1.6 (singlet peak of acetyl methyl protons in HA monomer). The HA–acrylate was stored at −20 °C until used.

*Microfluidic Device Design and Fabrication*: Water‐in‐oil flow focusing microfluidic molds were fabricated using soft lithography as previously described.^18^ In brief, KMPR 1025 or 1050 photoresist (Microchem) was used to create master molds on mechanical grade silicon wafers (University wafer). Manufacturer's suggestions were used for spinning photoresist to obtain a channel height of 55 µm. PDMS Sylgard 184 kit (Dow Corning) devices were made from the master molds. A 10:1 ratio of base to cross‐linker was used and added over the mold. Once degassed, the PDMS cured at 60 °C overnight. A glass microscope slide (VWR) and the PDMS molds were treated with oxygen plasma at 500 mTorr and 75 W for 15 s and pressed together to seal the channels. Immediately following channel sealing, Rain‐X was infused into the device and allowed to react for 20 min at room temperature. The Rain‐X was aspirated from the channels and the device was allowed to dry by air overnight.

*HA MAP Gel Formation, Purification, and Annealing*: The HA microgels were formed using a four inlet, one outlet microfluidic device previously reported.^20^ Briefly, two inlets were used for the “inner pinching” oil (1% v/v span‐80 in heavy mineral oil) and “outer” oil (5% v/v span‐80 in heavy mineral oil), while the other two inlets allowed the HA solution and the cross‐linker solution to be mixed immediately before the “pinching” occurred. The HA solution was freshly prepared by first dissolving HA–acrylate in 0.3 m triethanolamine (TEOA) pH 8.8 at 7% w/v. This solution was then used to dissolve three thiol‐containing pendent peptides: K‐peptide (Ac‐FKGGERCG‐NH2), Q‐peptide (Ac‐NQEQVSPLGGERCG‐NH2), and RGD at 500 × 10^−6^, 500 × 10^−6^, and 1000 × 10^−6^ m, respectively. The solution was then loaded into the 1 mL Hamilton Gastight Syringe after a 30 min incubation at 37 °C to prereact the thiol‐containing pendent peptides with the HA–acrylate. Meanwhile, the cross‐linker solution was prepared by dissolving the dithiol matrix metalloproteinase (MMP)‐sensitive linker peptide (Ac‐GCRDGPQGIWGQDRCG‐NH2, Genscript) in distilled water at 7.8 × 10^−3^ m followed by reacting with 10 × 10^−6^ m Alexa‐Fluor 488‐maleimide (LifeTechnologies) for green µgels, 10 × 10^−6^ m Alexa‐Fluor 555‐maleimide for red µgels, and 10 × 10^−6^ m Alexa‐Fluor 647‐maleimide for white µgels for 5 min. The cross‐linker solution was then loaded into another 1 mL Hamilton Gastight Syringe. Two syringe pumps were used to separately control the flow rates of the oils and the gel precursor solutions. The gel precursor solutions were coflowed at a 1:1 volume to form microgel droplets and left overnight at 25 °C to fully cross‐link. The final microgel composition was 3.5 wt% HA‐AM, 250 × 10^−6^ m K‐peptide, 250 × 10^−6^ m Q‐peptide, 500 × 10^−6^ m RGD, 5 × 10^−6^ m Alexa‐Fluor (488, 555, 647)‐maleimide, and 3.9 × 10^−3^ m cross‐linker (thiol:acrylamide (AM) is 0.8). The microgels were transferred to microcentrifuge tubes and HEPES buffer saline (pH 7.4 containing 10 × 10^−3^ m CaCl_2_) was added to each tube. The tubes were then centrifuged at 18 000 × *g* for 5 min to create a separation between the pelleted microgels and the oil and surfactant. The supernatant was aspirated and the procedure above was repeated until all the oil and surfactant was removed from the microgels (≈6 times). To anneal, pelleted microgels were mixed with 200 units mL^−1^ of activated FXIIIa and after injection, were incubated at 37 °C for 90 min to undergo a transglutaminase reaction between the K and Q peptides as previously described.^18^

*In Vitro Wound Healing Model*: Wound healing model molds were created by curing PDMS at 60 °C overnight. 2 cm × 4 cm rectangles were cut out of the PDMS and the resulting frames were bonded to a glass coverslip using plasma oxygen. 15 µL of green microgels mixed with 200 units mL^−1^ of FXIIIa was injected on one side of the mold. Immediately after, 15 µL of red microgels mixed with 200 units mL^−1^ of FXIIIa was injected in the middle and then 15 µL of white microgels mixed with 200 units mL^−1^ of FXIIIa was injected on the end. Scaffolds were allowed to anneal at 37 °C for 90 min and then imaged using a Nikon Ti Eclipse equipped with C2 laser light‐emitting diode (LED) excitation.

*In Vivo Wound Healing Model*: Animal procedures were performed in accordance with the US National Institutes of Health Animal Protection Guidelines and the University of California, Los Angeles (UCLA) Chancellor's Animal Research Committee. CLR:Skh1‐Hrhr mice (Charles River Laboratories) were anesthetized using aerosolized isofluorane (1.5 vol%) throughout the duration of the procedure. The skin was disinfected with sequential washes of povidone‐iodine and 70% ethanol. The mice were placed on their side and dorsal skin was pinched along the midline. A sterile 4 mm biopsy punch was used to create two clean‐cut, symmetrical, full‐thickness excisional wounds on either side of the dorsal midline. First, 10 µL of red µgels were added to the periphery of the skin wound using a positive displacement pipette (Gilson). Immediately after, 10 µL of green µgels were added in a circular motion inside the outer red layer to form the middle layer. Finally, 10 µL of white µgels were added to the center of the wound to form the inside layer. The mice were left under anesthesia for another 30 min to allow the hydrogel to anneal. Three days later, mice were sacrificed by isofluorane overdose and cervical dislocation. The skin samples were retrieved and cut in half through the wound and then imaged using a Nikon Ti Eclipse equipped with C2 laser LED excitation.

*In Vitro Subcutaneous and Stroke Models*: To create the molds, one 2 mm thick spacer was placed on each end of a coverslip. Another coverslip was placed on top of the spacers and clipped together using a small paper clip on each end. Using a positive displacement pipette (Gilson), 15 µL of white µgels were pulled up. The pipette volume was then changed to 30 µL and 15 µL of green µgels were pulled up. Finally, the pipette volume was changed to 45 µL and 15 µL of red µgels were pulled up, resulting in 45 µL of MAP gel in the pipette. Next, the gel was transferred to a 100 µL Hamilton Syringe by back‐loading to maintain the same pattern. For the subcutaneous injections, a controlled syringe dispenser (Hamilton) was used to inject the entire 45 µL MAP hydrogel between the two coverslips. The resulting MAP gel was allowed to anneal at 37 °C for 90 min and then imaged using a Nikon Ti Eclipse equipped with C2 laser LED excitation. For the stroke injections, the 100 µL syringe was loaded onto a Nexus 3000 syringe pump (Chemyx) and injected between two coverslips at a 1 µL min^−1^ injection rate. The resulting MAP gel was allowed to anneal at 37 °C for 90 min and then imaged using a Nikon Ti Eclipse equipped with C2 laser LED excitation.

*In Vivo Subcutaneous Implant Model*: All in vivo studies were conducted in compliance with the National Institutes of Health(NIH) Guide for Care and Use of Laboratory Animals and UCLA ARC standards. Similar to the in vitro procedure, using a positive displacement pipette (Gilson), 15 µL of white µgels were pulled up. The pipette volume was then changed to 30 µL and 15 µL of green µgels were pulled up. Finally, the pipette volume was changed to 45 µL and 15 µL of red µgels were pulled up, resulting in 45 µL of MAP gel in the pipette. Next, the gel was transferred to a 100 µL Hamilton Syringe by back‐loading to maintain the same pattern. A controlled syringe dispenser (Hamilton) was used to inject the entire 45 µL MAP hydrogel subcutaneously into the mice. The mice were left under anesthesia for another 30 min to allow the hydrogel to anneal. Three days later, mice were sacrificed by isofluorane overdose and cervical dislocation. The skin samples were retrieved and imaged using a Nikon Ti Eclipse equipped with C2 laser LED excitation.

*In Vivo Photothrombotic Stroke Model*: Animal procedures were performed in accordance with the US National Institutes of Health Animal Protection Guidelines and the University of California Los Angeles Chancellor's Animal Research Committee. A cortical photothrombotic stroke was induced on 8–12week male C57BL/6 mice obtained from Jackson laboratories (Bar Harbor, ME). The mice were anesthetized with 2.5% isoflurane and placed onto a stereotactic setup. The mice were kept at 2.5% isoflurane in N_2_O:O_2_ for the duration of the surgery. A midline incision was made and Rose Bengal (10 mg mL^−1^, Sigma‐Aldrich) was injected intraperitoneally at 10 µL g^−1^ of mouse body weight. After 5 min of Rose Bengal injection, a 2 mm diameter cold fiberoptic light source was centered at 0 mm anterior/1.5 mm lateral left of the bregma for 18 min and a burr hole was drilled through the skull in the same location. All mice were given sulfamethoxazole and trimethoprim oral suspension (TMS, 303 mL TMS per 250 mL H_2_O, Amityville, NY) every five days for the entire length of the experiment. Using a positive displacement pipette (Gilson), 2 µL of white µgels were pulled up. The pipette volume was then changed to 4 µL and 2 µL of green µgels were pulled up. The pipette volume was then changed to 6 µL and 2 µL of red µgels were pulled. This was repeated once more to create a total volume of 12 µL. Next, the gel was transferred to a 25 µL Hamilton Syringe by back‐loading to maintain the same pattern. Five days poststroke, the Hamilton Syringe containing the patterned gel was connected to a pump and 6 µL of MAP was injected into the stroke cavity using a 30 gauge needle at a depth of 0.8 mm and the same stereotaxic coordinates as above at an infusion speed of 1 µL min^−1^. The needle was withdrawn from the mouse brain 5 min after the injection to allow for annealing of the microgels. Ten days postinjection, mice were sacrificed via transcardial perfusion of 1× phosphate buffered saline (PBS) followed by 40 mL of 4% paraformaldehyde (PFA). The brains were isolated and postfixed in 4% PFA overnight and submerged in 30% w/v sucrose solution for 48 h. Tangential cortical sections of 100 µm thickness were sliced using a cryostat until halfway into the stroke. The remaining half of the brain was unmounted and imaged using a Nikon Ti Eclipse equipped with C2 laser LED excitation.

*Image Analysis Using ImageJ Software*: All images were analyzed using ImageJ software. In each image, three regions were traced to separate the white, red, and green regions. Each region was analyzed by obtaining the percent area occupied by the white, red, and green µgels. Moreover, each µgel color was analyzed to obtain the percent distribution of the µgel color in each region.

## Conflict of Interest

The authors declare no conflict of interest.
